# Nutritional-inflammatory indices optimize the diagnostic performance of FIB-4 for advanced fibrosis/cirrhosis in patients with benign liver disease

**DOI:** 10.1080/07853890.2026.2639649

**Published:** 2026-03-13

**Authors:** Jing Zhou, Daofeng Yang, Jun Lu

**Affiliations:** ^a^ Department of Medical Oncology, Beijing YouAn Hospital, Capital Medical University, Beijing, China; ^b^ Laboratory for Clinical Medicine, Capital Medical University, Beijing, China; ^c^ Tongji Medical College, Huazhong University of Science and Technology, Tongji Hospital, Wuhan, China

**Keywords:** Nutritional-inflammatory indices, advanced fibrosis/cirrhosis, PAR, PNI, FIB-4, benign liver diseases

## Abstract

**Background/objectives:**

To address the high false-positive rate of fibrosis-4 (FIB-4), we hypothesize that combining it with readily available nutritional-inflammatory indices can improve the diagnostic accuracy for advanced liver fibrosis/cirrhosis.

**Methods:**

Diagnostic performance was sequentially evaluated: first, individual metrics and FIB-4 were compared *via* receiver operating characteristics (ROC) curve analysis; then, their incremental value was assessed using DeLong’s test, net reclassification improvement (NRI) and integrated discrimination improvement (IDI); finally, the clinical utility of four combined strategies was examined.

**Results:**

In discriminating advanced fibrosis/cirrhosis, the platelet-to-albumin ratio (PAR) showed a significantly higher area under the curve (AUC) of 0.698 than the haemoglobin, albumin, lymphocyte and platelet (HALP, AUC= 0.494; *p* < .001). Similarly, the prognostic nutritional index (PNI) also outperformed HALP in diagnostic performance (AUC = 0.658; *p* < .0001). Adding PAR or PNI to FIB-4 significantly improved diagnostic performance, as evidenced by Delong’s test, NRI and IDI. Specifically, the combination of FIB-4 and PAR improved specificity (88.5% vs 38.5%) and overall accuracy (63.7% vs 54.8%) compared to FIB-4 alone, while the FIB-4 and PNI combination achieved a specificity of 80.3%. The parallel strategy (FIB-4 + ‘PAR or PNI’) maintained a sensitivity of 61.9% and achieved the highest negative predictive value (65.2%). The serial strategy (FIB-4 + ‘PAR and PNI’) provided the highest specificity (95.1%) and positive predictive value (81.8%).

**Conclusions:**

In scenarios prioritizing high sensitivity, FIB-4 alone is suitable. For high specificity, a sequential strategy (FIB-4 + ‘PAR and PNI’) is recommended. For balanced performance, a parallel strategy (FIB-4 + ‘PAR or PNI’) provides optimal feasibility.

## KEY MESSAGES

In settings where high sensitivity is prioritized for screening, the FIB-4 index may be used alone to minimize the risk of missed diagnoses.

In settings where high specificity is the primary goal, a sequential strategy (FIB-4 + ‘PAR and PNI’) is recommended to reduce the false positive rate as much as possible.

For routine screening that requires balanced overall diagnostic performance, a parallel strategy (FIB-4 + ‘PAR or PNI’) demonstrates relatively even performance across metrics and offers good feasibility for clinical application.

## 1. Introduction

Cirrhosis is a global public health issue with complex and diverse aetiologies, primarily attributed to metabolic dysfunction-associated steatotic liver disease, viral hepatitis (especially hepatitis B and C) and alcoholic liver disease [1]. The disease spectrum encompasses a continuum from the compensated stage, characterized by the absence of apparent symptoms and preserved hepatic function sufficient to meet physiological demands, to the decompensated stage, marked by severe complications such as ascites, variceal haemorrhage and hepatic encephalopathy [2]. Although disease staging demonstrates significant differences in prognosis and therapeutic management, it is undeniable that all patients with cirrhosis remain in a high-risk state, facing potential threats including hepatic failure, complications of portal hypertension and hepatocellular carcinoma [3–5].

As described above, cirrhosis is a progressive pathological process triggered by diverse aetiologies. Despite variations in the initial injury mechanisms, disease progression ultimately converges on two interrelated, overarching core pathways: ‘systemic inflammation’ and ‘malnutrition’ [6,7]. Persistent liver injury activates inflammatory cascades and impairs hepatic synthetic and metabolic functions, leading to nutrition-immune dysfunction characterized by hypoalbuminemia, lymphopenia and other manifestations. This dysfunction not only serves as a marker of disease severity but also directly participates in and drives the initiation and progression of liver fibrosis [8,9].

Nutritional-inflammatory indices, represented by the PNI, PAR and HALP, integrate routine haematological parameters such as albumin, lymphocyte count, platelets and haemoglobin. These metrics collectively reflect a patient’s nutritional status, inflammatory burden and hepatic synthetic function, thereby offering a more comprehensive insight into the long-term impact of the disease and the overall pathophysiological milieu. They have demonstrated significant value in the assessment of the onset and prognosis of various diseases [10–14]. In cirrhosis, ongoing nutritional depletion and persistent low-grade inflammation continuously drive disease progression, suggesting that such indices may possess unique potential for identifying early-stage or subclinical cirrhosis.

FIB-4, a non-invasive index based on routine haematological parameters, is recommended by both domestic and international guidelines as a tool for initial screening of liver fibrosis risk in community and high-risk populations [15,16]. However, in clinical practice, the FIB-4 index alone has limitations, including insufficient specificity and a tendency for false-positive results [17]. This creates a challenge: if individuals with a positive FIB-4 screen are referred for confirmatory testing based solely on this result, a proportion of patients without advanced liver fibrosis or cirrhosis will inevitably undergo unnecessary confirmatory examinations, such as vibration-controlled transient elastography. This not only increases healthcare costs but may also cause unnecessary patient anxiety. Therefore, optimizing referral decisions for FIB-4-positive individuals is of critical importance.

To address unnecessary referrals arising from the limited specificity and high false-positive rate of using FIB-4 alone, it is crucial to evaluate and integrate supplementary indicators to establish a flexible, scenario-adapted combined diagnostic strategy for the precise and efficient identification of advanced liver fibrosis/cirrhosis. Given that nutritional-inflammatory indices are readily available, cost-effective and closely linked to the pathophysiology of hepatic fibrosis progression, we hypothesize that combining them with FIB-4 could optimize its diagnostic performance.

So, we systematically evaluated and compared the diagnostic performance of nutritional-inflammatory indices, FIB-4 alone and their combinations in patients with benign liver disease. We not only quantified the incremental value of these indices through statistical analyses, but also assessed the applicability of different combination strategies in specific clinical scenarios, such as high-sensitivity screening versus high-specificity confirmation. The aim was to clarify the practical utility and relative merit of these integrated approaches in different clinical contexts, thereby providing evidence for the development of more targeted non-invasive diagnostic pathways.

## 2. Materials and methods

### 2.1. Participants

This study is a cross-sectional investigation conducted within a single-centre cohort of patients with benign liver disease. Patients with established liver disease were selected as the study population because this group represents a core population at high risk for progression to liver fibrosis/cirrhosis and their clinical data can be systematically obtained within the hospital setting. Inclusion criteria: (1) presentation with liver disease or related symptoms; (2) ≥18 years of age; (3) availability of liver imaging or liver histopathological examination results. Exclusion criteria: (1) decompensated cirrhosis; (2) presence of liver malignancy or malignancy of other organs; (3) heart failure; (4) lung failure; (5) chronic kidney disease; or (6) any missing data. We screened patients who visited the Tongji Hospital, Tongji Medical College, Huazhong University of Science and Technology between 1 January 2021 and 1 January 2022. The relevant information was gathered between February and July 2024. Finally, a total of 292 patients who met the above criteria were ultimately included in the study. The final data analysis was completed in September 2025.

The determination of advanced fibrosis/cirrhosis in participants was primarily based on histopathological examination of liver tissue (available for 32 patients in this study); for the remaining patients, the diagnosis was made by comprehensive assessment of imaging findings and clinical manifestations. The imaging modalities utilized included ultrasound, contrast-enhanced computed tomography or magnetic resonance imaging. For each of these modalities, the interpretation of findings strictly adhered to their respective, widely accepted diagnostic criteria for liver fibrosis and cirrhosis [18]. Based on these diagnostic findings, patients were categorized into two groups: those with advanced fibrosis/cirrhosis, defined either by histopathological evidence of significant fibrosis or cirrhosis or, in the absence of liver biopsy, by imaging characteristics such as an irregular liver surface and lobar disproportion; and those with non-advanced fibrosis/cirrhosis, which included patients with no histopathological evidence of significant fibrosis or cirrhosis or, when biopsy was not available, imaging findings indicating a smooth liver surface and normal lobar architecture.

### 2.2. Calculation formula of indices

The calculation formulas for the nutritional-inflammatory indices and conventional liver fibrosis markers are provided in Supplemental Table 1.

### 2.3. Statistical analysis

The statistical analyses were performed using R software (version 4.5.0). Statistical significance was defined as a two-tailed *p* value < .05. Categorical data are presented as numbers and percentages and compared using the chi-square test. Continuous variables with non-normal distribution are expressed as median (interquartile range) and between-group differences were analysed using the Mann–Whitney *U* test.

Univariable and multivariable logistic regression models were employed to evaluate the associations of the nutritional-inflammatory indices (PAR, PNI, HALP) and the non-invasive liver fibrosis marker (FIB-4) with the presence of liver cirrhosis. Three models were constructed for each indicator: a crude model with no adjustments (Model 1), a model adjusted for sex and age (Model 2) and a fully adjusted model that further included the aetiology of liver disease (Model 3).

The diagnostic performance was evaluated sequentially. First, the individual performance of the nutritional-inflammatory indices and FIB-4 was assessed by comparing their ROC curves and then calculating clinical performance metrics (e.g. sensitivity, specificity) based on established cut-offs. Next, the incremental value of adding these indices to the FIB-4 model was tested using DeLong’s test, NRI and IDI. Finally, the performance of four specific combined strategies was evaluated using clinical cut-offs.

## 3. Results

### 3.1. Characteristics of participants

A total of 292 patients with benign liver diseases were initially screened. Among these, 32 cases were biopsy-confirmed, including 6 patients with advanced fibrosis/cirrhosis and 26 with non-advanced fibrosis/cirrhosis. The overall aetiological distribution was as follows: 196 patients with chronic hepatitis B, 12 with chronic hepatitis C, 19 with alcohol-related liver disease, 21 with autoimmune liver disease, 36 with drug-induced liver injury, 5 with hepatic haemangioma and 3 with benign liver nodules.

We focused our primary analysis on 248 patients at risk of progressive fibrosis/cirrhosis (at-risk cohort), comprising 126 with advanced fibrosis/cirrhosis and 122 without. Within this at-risk cohort, 19 cases were biopsy-confirmed (6 patients with advanced fibrosis/Cirrhosis and 13 with non-advanced fibrosis/Cirrhosis). The aetiological distribution was: 196 patients with chronic hepatitis B, 12 with chronic hepatitis C, 19 with alcohol-related liver disease and 21 with autoimmune liver disease. Additionally, 44 patients with conditions considered to have minimal risk for progression to liver fibrosis or cirrhosis (specifically, 36 with drug-induced liver injury, 5 with hepatic haemangioma and 3 with benign liver nodules) were included in the initial screening. Among these 44 patients, 13 cases were biopsy-confirmed. Their data were retained for a subsequent sensitivity analysis to assess the robustness of our findings across a broader, real-world diagnostic spectrum.

Within the at-risk cohort, we found no significant differences in gender between patients with advanced fibrosis/cirrhosis and those without. However, it is noteworthy that the average age of patients with advanced fibrosis/cirrhosis was significantly higher than that of patients without (Table 1). Among the 3 nutritional-inflammatory indices, all except for HALP score showed significant differences between patients with advanced fibrosis/cirrhosis and those without. Furthermore, we evaluated the non-invasive biomarker FIB-4 for the identification of advanced liver fibrosis/cirrhosis. Notably, FIB-4 demonstrated a statistically significant difference between advanced fibrotic/cirrhotic and non-advanced fibrotic/cirrhotic patients (Table 1 and Supplemental Figure 1).

**Table 1.** Characteristics of participants.

Variable	Non-advanced fibrosis/cirrhosis (*N* = 122)	Advanced fibrosis/cirrhosis (*N* = 126)	*p* Value
Age (years)	46.0 (32.0–55.0)	55.0 (45.0–61.0)	<.001
Gender			.157
Male	101 (82.8%)	94 (74.6%)	
Female	21 (17.2%)	32 (25.4%)	
Aetiology			<.001
Hepatitis B	110 (90.2%)	86 (68.3%)	
Hepatitis C	3 (2.5%)	9 (7.1%)	
Other	9 (7.4%)	31 (24.6%)	
WBC^a^ (×10⁹/L)	5.3 (4.3–7.0)	5.0 (3.5–6.2)	.031
Neutrophil (×10⁹/L)	3.2 (2.5–4.5)	3.0 (2.2–4.3)	.119
Lymphocyte (×10⁹/L)	1.3 (1.0–1.7)	1.0 (0.7–1.4)	<.001
Monocyte (×10⁹/L)	0.5 (0.4–0.7)	0.5 (0.3–0.7)	.357
Platelet (×10⁹/L)	134.5 (99.5–175.5)	85.5 (60.0–121.8)	<.001
Haemoglobin (g/L)	127.5 (107.0–143.8)	120.0 (104.0–133.0)	.009
ALT^b^ (U/L)	75.0 (32.0–119.5)	64.0 (39.2–153.2)	.222
AST^c^ (U/L)	82.5 (44.0–152.0)	43.5 (23.0–156.8)	.010
Albumin (g/L)	36.0 (32.6–40.7)	32.7 (29.0–38.2)	<.001
TBIL^d^ (μmol/L)	133.5 (29.7–310.9)	63.4 (21.9–230.1)	.140
DBIL^e^ (μmol/L)	115.0 (17.2–239.9)	39.5 (11.2–188.0)	.084
ALP^f^ (U/L)	126.0 (95.8–148.5)	116.5 (92.5–150.0)	.466
γ-GT^g^ (U/L)	121.5 (63.2–215.8)	71.5 (32.2–117.5)	<.001
PT^h^ (s)	15.7 (13.8–20.6)	17.8 (15.4–21.8)	<.001 .004
APTT^i^ (s)	40.8 (37.9–46.8)	44.2 (39.6–50.9)	.011
PNI^j^	43.3 (38.1–49.1)	38.0 (34.6–44.8)	<.001
PAR^k^	3.8 (2.8–4.8)	2.6 (1.7–3.7)	<.001
HALP^l^	49.2 (30.5–67.7)	47.6 (31.3–65.8)	.867
FIB-4^m^	3.3 (2.1–4.9)	4.2 (2.3–7.9)	.017

^a^White blood cell.

^b^Alanine aminotransferase.

^c^Aspartate aminotransferase.

^d^Total bilirubin.

^e^Direct bilirubin.

^f^Alkaline phosphatase.

^g^γ-Glutamyl transpeptidase.

^h^Prothrombin time.

^i^Activated partial thromboplastin time.

^j^Prognostic nutritional index.

^k^Platelet-to-albumin ratio.

^l^Haemoglobin, albumin, lymphocyte and platelet.

^m^Fibrosis-4.

### 3.2. Logistic regression analysis of cirrhosis-associated factors

After adjustment for age, sex and aetiology, most indices retained significant independent associations with advanced fibrosis/cirrhosis (Table 2). Specifically, the PNI demonstrated a significant negative association (OR = 0.939, 95% CI: 0.903–0.976, *p* = .002). Similarly, the PAR was significantly associated with a lower risk (OR = 0.682, 95% CI: 0.554–0.827, *p* < .001). Higher levels of FIB-4 (OR = 1.069, 95% CI: 1.004–1.148, *p* = .049) were also associated with an increased risk of advanced fibrosis/cirrhosis. In contrast, the association for HALP was not statistically significant (OR = 1.003, 95% CI: 0.994–1.012, *p* = .571).

**Table 2.** Logistic regression analysis of independent factors associated with advanced fibrosis/cirrhosis.

Model	Variable	OR^h^	95%CI^i^	*p* Value
Model 1^a^				
	PNI^d^	0.934	0.900–0.966	<.0001
	PAR^e^	0.646	0.534–0.771	<.0001
	HALP^f^	1.004	0.997–1.012	.281
	FIB-4^g^	1.091	1.032–1.165	.005
Model 2^b^				
	PNI	0.951	0.915–0.986	.007
	PAR	0.688	0.563–0.828	.0001
	HALP	1.005	0.997–1.014	.234
	FIB-4	1.056	0.994–1.130	.095
Model 3^c^				
	PNI	0.939	0.903–0.976	.002
	PAR	0.682	0.554–0.827	.0002
	HALP	1.003	0.994–1.012	.571
	FIB-4	1.069	1.004–1.148	.049

^a^Unadjusted.

^b^Adjusted for age and gender.

^c^Adjusted for age, gender and aetiology.

^d^Prognostic nutritional index.

^e^Platelet-to-albumin ratio.

^f^Haemoglobin, albumin, lymphocyte and platelet.

^g^Fibrosis-4.

^h^Odds ratio.

^i^Confidence interval.

### 3.3. Diagnostic performance of nutritional-inflammatory indices: ROC curves

**Figure 1.**
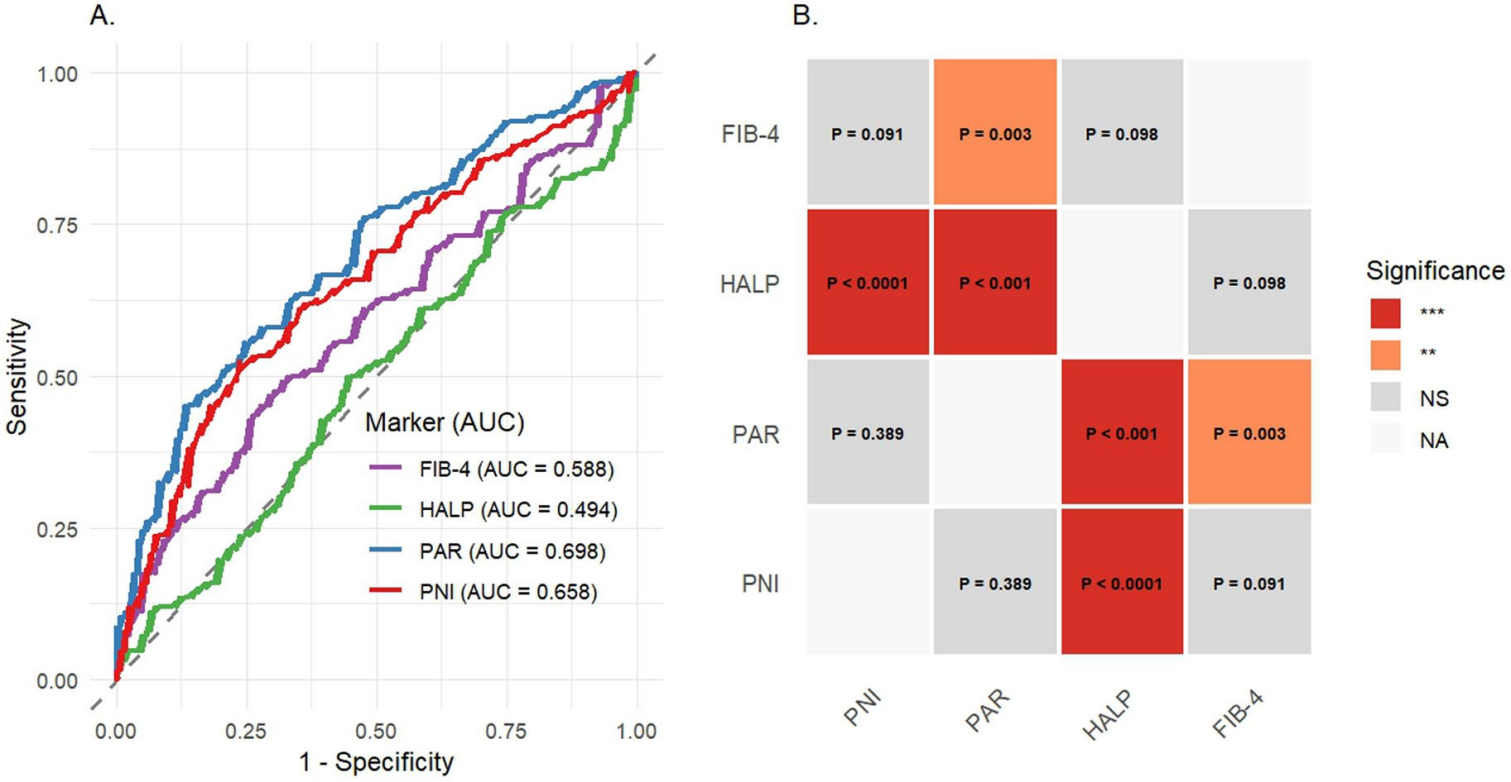
ROC and DeLong test of nutritional-inflammatory indices for advanced fibrosis/cirrhosis diagnosis. (A) ROC curves and (B) heatmap of DeLong test results.

To evaluate their diagnostic performance, we constructed ROC curves for the nutritional-inflammatory indices using the presence of advanced fibrosis/cirrhosis as the outcome and calculated the AUC (Supplemental Table 2). The AUC values for discriminating advanced fibrosis/cirrhosis were 0.698 (95% CI: 0.633–0.763) for PAR, 0.658 (95% CI: 0.590–0.726) for PNI and 0.494 (95% CI: 0.422–0.566) for HALP. Pairwise comparisons revealed that while the AUC of PAR was not significantly different from that of PNI (*p* = .389), both PAR and PNI demonstrated statistically superior discrimination compared to HALP (*p* < .001 and *p* < .0001, respectively). Furthermore, we compared the diagnostic performance of these nutritional-inflammatory indices with that of the non-invasive marker for advanced fibrosis/cirrhosis, FIB-4, by evaluating their AUCs, as detailed in Figure 1.

### 3.4. Evaluation of diagnostic performance based on predefined risk stratification

**Figure 2.**
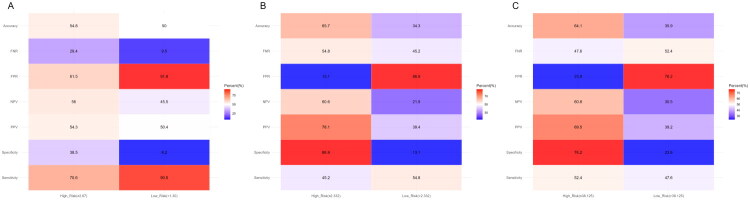
The diagnostic performance of FIB-4, PAR and PNI. (A) Heatmap of diagnostic performance metrics for FIB-4; (B) heatmap of diagnostic performance metrics for PAR and (C) heatmap of diagnostic performance metrics for PNI.

We analysed the prevalence of advanced liver fibrosis/cirrhosis across different risk stratifications based on the FIB-4, PAR and PNI and compared their diagnostic performance. The results indicated that the PAR, in its high-risk group (≤2.332), demonstrated a low sensitivity (45.2%) and relatively high specificity (86.9%), with a moderate PPV of 78.1% and an overall diagnostic accuracy of 65.7%. However, its low-risk group (>2.332) exhibited a moderate FNR of 45.2% (Figure 2(B)). The PNI, in its high-risk group (≤38.125), showed favourable specificity (76.2%) and a moderate PPV of 69.5%, with an overall accuracy of 64.1%; yet, its low-risk group (>38.125) also had a considerable FNR of 52.4% (Figure 2(C)). In contrast, the FIB-4, in its high-risk group (≥2.67), displayed high sensitivity (70.6%) but relatively low specificity (38.5%). Notably, its low-risk group (<1.30) achieved a markedly low FNR of only 9.5% (Figure 2(A)).

### 3.5. Incremental value of PAR and PNI to the FIB-4 model

**Figure 3.**
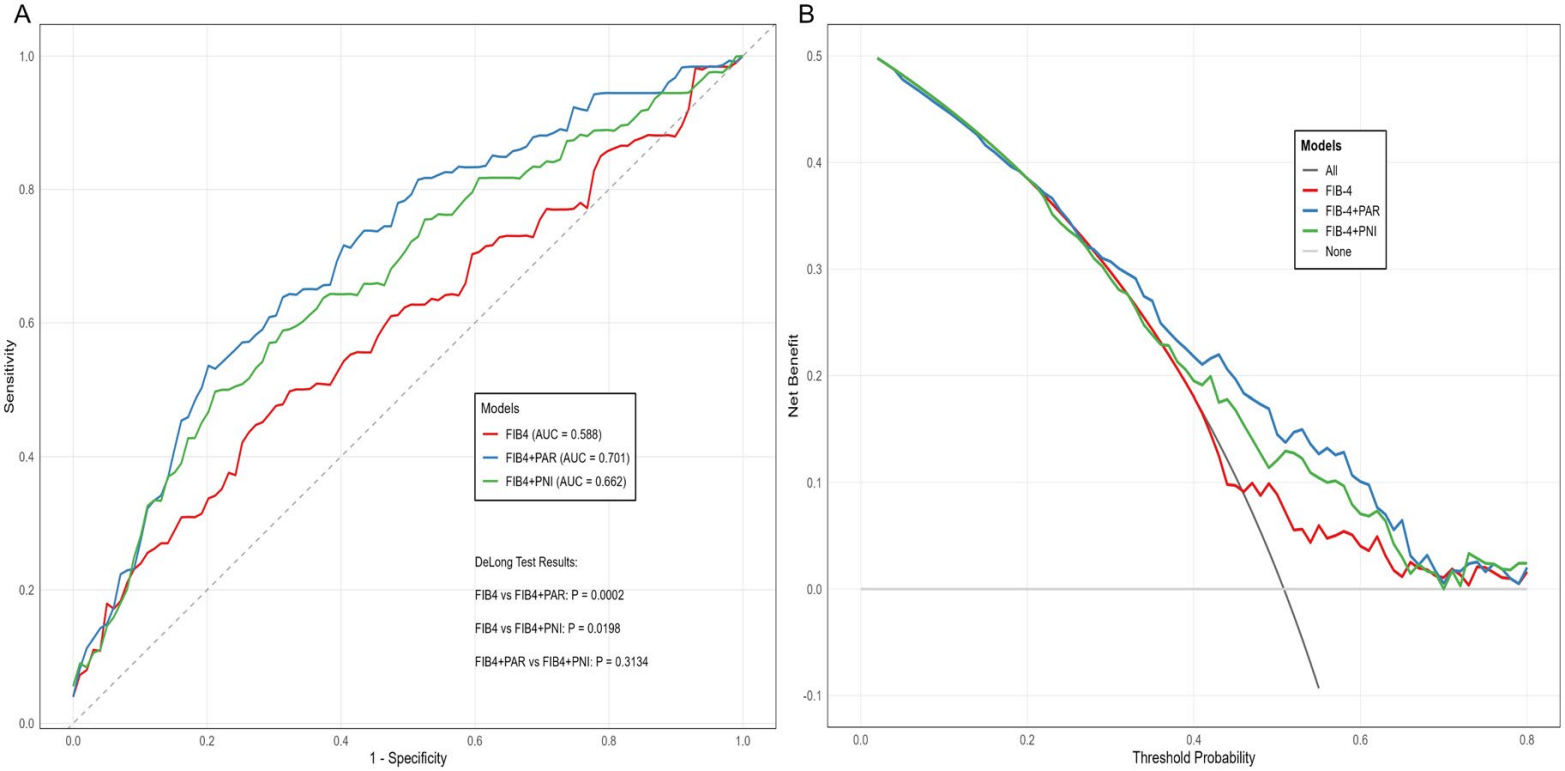
Incremental value of PAR in the diagnostic performance of FIB-4 and APRI. (A) ROC curves of models and (B) DCA of models.

We systematically evaluated the incremental diagnostic value of adding the nutritional-inflammatory index PAR and PNI to FIB-4 for advanced fibrosis/cirrhosis detection. As shown in Table 3, the addition of PAR to the FIB-4 significantly improved diagnostic performance (DeLong’s test: *p* < .001; NRI = 0.0998, *p* < .0001; IDI = 0.0888, *p* < .0001). A similar improvement trend was observed when PNI was added to the FIB-4, though the improvement was more modest (DeLong’s test: *p* = .020; NRI = 0.0583, 95% CI: NA; IDI = 0.0399, *p* < .001). The corresponding ROC curves (Figure 3(A)) and DCA (Figure 3(B)) curves visually demonstrated the enhancement in model discrimination and clinical net benefit, respectively.

**Table 3.** Comparison of diagnostic performance of FIB-4 and APRI with and without PAR.

	DeLong test			NRI^e^ test			IDI^f^ test		
Model	Difference between area	95%CI^d^	*p* Value	Categorical	95%CI	*p* Value	Categorical	95%CI	*p* Value
Total									
PAR^a^+FIB-4 vs. FIB-4^b^	0.1130	0.0534, 0.1726	<.001	0.1072	0.0747, 0.1397	<.0001	0.0741	0.0430, 0.1053	<.0001
PNI^c^+FIB-4 vs. FIB-4	0.0739	0.0117, 0.1361	.020	0.0583	–	NA^g^	0.0399	0.0160, 0.0639	.001
Male (gender subgroup analysis)									
PAR + FIB-4 vs. FIB-4	0.1065	0.0422, 0.1708	.001	0.1768	0.1114, 0.2423	<.0001	0.0717	0.0375, 0.1058	<.0001
PNI+FIB-4 vs. FIB-4	0.0624	−0.0006, 0.1253	.052	0.1244	0.0701, 0.1787	<.0001	0.0334	0.0086, 0.0583	.008
Female (gender subgroup analysis)									
PAR+FIB-4 vs. FIB-4	0.1622	0.0125, 0.3119	.034	0.1935	0.0653, 0.3216	.003	0.0962	0.0126, 0.1797	.024
PNI+FIB-4 vs. FIB-4	0.1399	−0.0434, 0.3232	.135	0.1935	0.0653, 0.3216	.003	0.0707	0.0004, 0.1409	.049
Age ≥60 years (age subgroup analysis)									
PAR+FIB-4 vs. FIB-4	0.1601	−0.1298, 0.4499	.280	0.0873	−0.0500, 0.2246	.213	0.0578	−0.0039, 0.1196	.067
PNI+FIB-4 Vs. FIB-4	0.0026	−0.0064, 0.0117	.566	0	0	NA	0	−0.0003, 0.0003	.975
Age < 60 years (age subgroup analysis)									
PAR+FIB-4 vs. FIB-4	0.1239	0.0490, 0.1987	.001	0.1777	0.1055, 0.2498	<.0001	0.0682	0.0333, 0.1032	.0001
PNI+FIB-4 vs. FIB-4	0.0999	0.0214, 0.1785	.013	0.1300	0.0659, 0.1942	.0001	0.0531	0.0206, 0.0856	<.01
Hepatitis B (aetiology subgroup analysis)									
PAR+FIB-4 vs. FIB-4	0.0952	0.0335, 0.1570	.003	0.1315	0.0625, 0.2005	<0.001	0.0636	0.0308, 0.0965	.0001
PNI+FIB-4 vs. FIB-4	0.0659	0.0048, 0.1269	.035	0.1057	0.0419, 0.1695	0.001	0.0426	0.0148, 0.0703	.003
Hepatitis C (aetiology subgroup analysis)									
PAR+FIB-4 vs. FIB-4	0.1852	−0.5251, 0.8955	.609	0.2222	−0.2593, 0.7037	0.366	0.1673	−0.1248, 0.4594	.262
PNI+FIB-4 vs. FIB-4	0.2222	−0.3775, 0.8219	.468	0.2222	−0.2593, 0.7037	0.366	0.2179	−0.0124, 0.4482	.064
Other (aetiology subgroup analysis)									
PAR+FIB-4 vs. FIB-4	0.2366	−0.0348, 0.5079	.088	0.0789	−0.1162, 0.2739	0.428	0.0745	−0.0243, 0.1734	.139
PNI+FIB-4 vs. FIB-4	0.1577	−0.1704, 0.4858	.346	0	0	NA	0.0381	−0.0327, 0.1089	.292

^a^Platelet-to-albumin ratio.

^b^Fibrosis-4.

^c^Prognostic Nutritional Index.

^d^Confidence interval.

^e^Net reclassification improvement.

^f^Integrated discrimination improvement.

^g^Not available.

**Figure 4.**
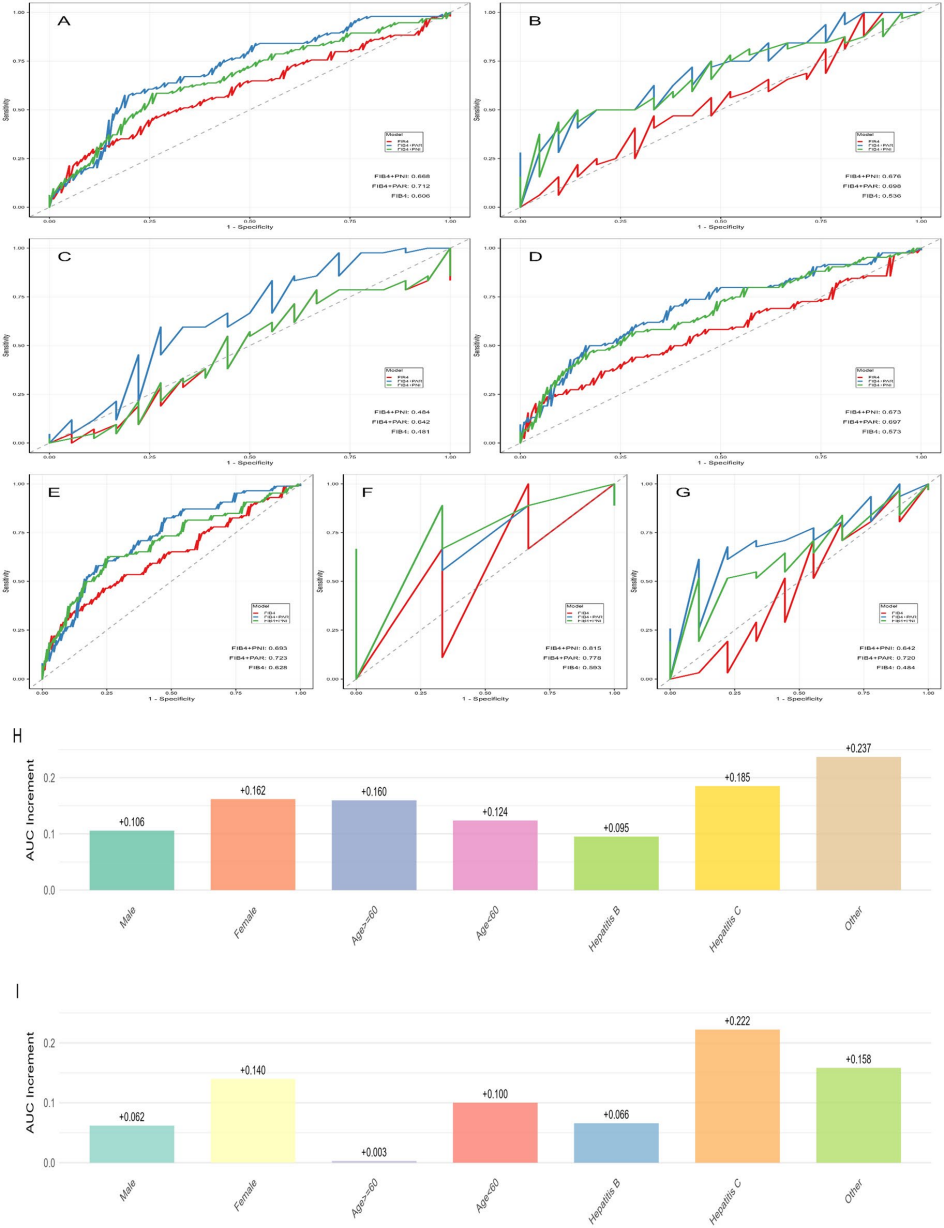
Subgroup analysis of the incremental value of PAR and PNI added to FIB-4 for advanced fibrosis/cirrhosis diagnosis. (A) Male subgroup; (B) female subgroup; (C) age ≥ 60 years subgroup; (D) age < 60 years subgroup; (E) hepatitis B aetiology subgroup; (F) hepatitis C aetiology subgroup; (G) other aetiologies subgroup; (H) incremental AUC of PAR + FIB-4 compared to FIB-4 alone and (I) incremental AUC of PNI + FIB-4 compared to FIB-4 alone.

To verify the generalizability of this incremental value, we conducted subgroup analyses. As summarized in Table 3, the addition of PAR and PNI consistently improved diagnostic performance across most subgroups. The corresponding ROC curves provided a visual representation of this stable improvement trend in the gender (Figure 4(A,B)), age (Figure 4(C,D)) and aetiology (Figure 4(E–G)) subgroups, with the exception of Hepatitis C patients. The incremental AUC values of PAR and PNI added to FIB-4 are visualized using bar charts in Figure 4(H,I), respectively.

### 3.6. Diagnostic performance of combined strategies using predefined cut-offs

**Figure 5.**
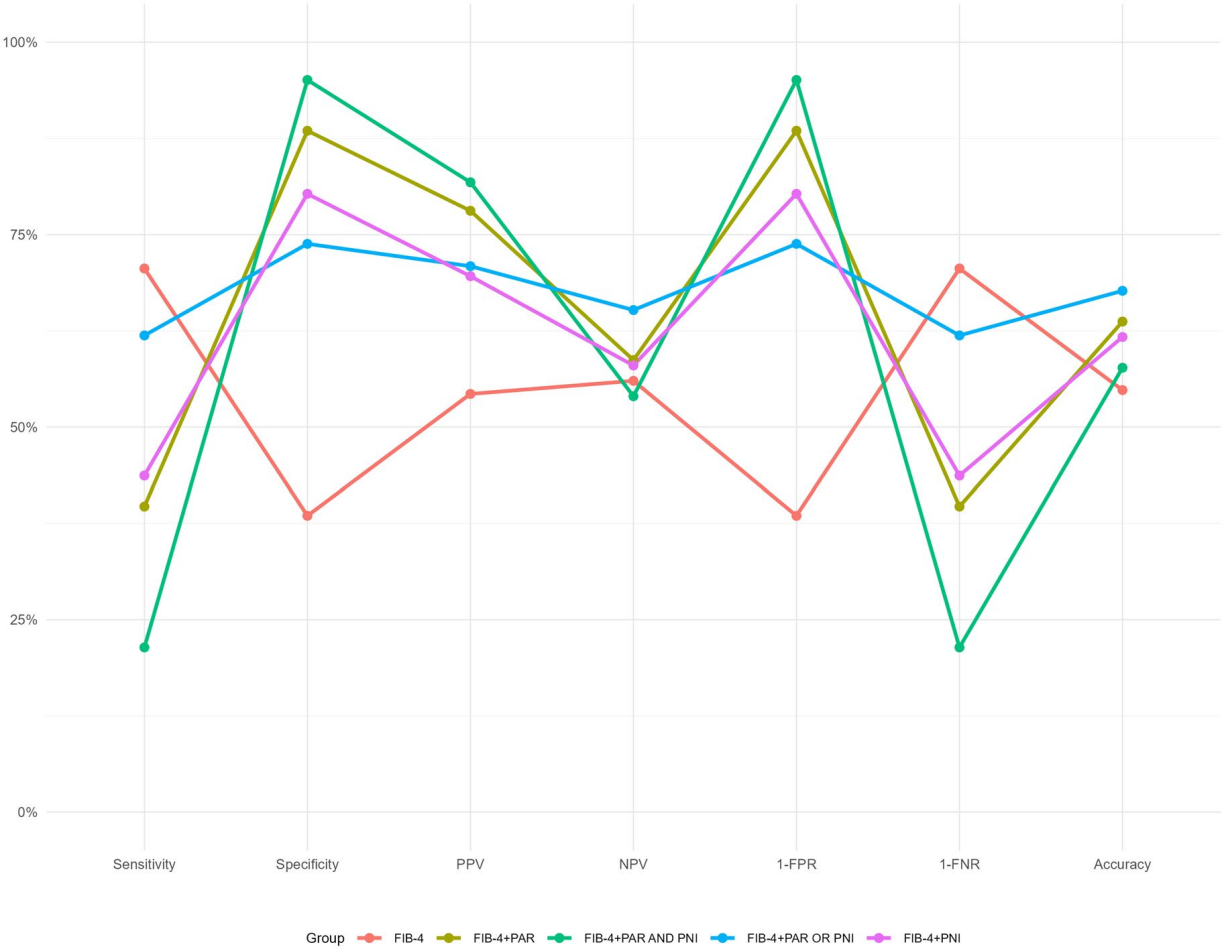
The enhancement of advanced liver fibrosis/cirrhosis diagnostic performance by combining PAR and PNI with FIB-4.

We next assessed the performance of specific diagnostic strategies that combined FIB-4 with PAR and/or PNI using predefined cut-offs. The results demonstrated that, compared to using FIB-4 alone, the combined strategies significantly optimized multiple diagnostic metrics (Figure 5). Specifically, the combination of FIB-4 and PAR showed notable improvements in specificity (88.5% vs 38.5%), positive predictive value (78.1% vs 54.3%) and overall accuracy (63.7% vs 54.8%). The FIB-4 and PNI combination further increased specificity to 80.3%, with a corresponding decrease in sensitivity to 43.7% and an accuracy of 61.7%. The parallel strategy (FIB-4 + ‘PAR or PNI’) maintained relatively high sensitivity (61.9% vs 70.6%) while achieving balanced specificity (73.8%) and the highest negative predictive value (65.2%). In contrast, the serial strategy (FIB-4 + ‘PAR and PNI’) achieved the highest specificity (95.1%) and positive predictive value (81.8%), but at the cost of a significant reduction in sensitivity (21.4%).

### 3.7. Sensitivity analysis

To assess the robustness of our findings, we performed a sensitivity analysis by including all 292 initially screened patients. The baseline characteristics of the full cohort are presented in Supplemental Table 3. We analysed the prevalence of advanced liver fibrosis/cirrhosis across different risk stratifications based on FIB‑4 (Supplemental Figure 2A), PAR (Supplemental Figure 2B) and PNI (Supplemental Figure 2C) in the full cohort. Then, we systematically evaluated the incremental diagnostic value of adding the nutritional-inflammatory index PAR and PNI to FIB-4 for cirrhosis detection in the full cohort. As shown in Table 4, in the overall population, the addition of PAR to the FIB-4 significantly improved diagnostic performance (DeLong’s test: *p* < .0001; NRI = 0.1890, *p* < .0001; IDI = 0.1122, *p* < .0001). A similar improvement trend was observed when PNI was added to the FIB-4 (DeLong’s test: *p* = .003; NRI = 0.1236, *p* < .0001; IDI = 0.0513, *p* < .0001).

**Table 4.** Comparison of diagnostic performance of FIB-4 and APRI with and without PAR in the full cohort.

	DeLong test			NRI^e^ test			IDI^f^ test		
Model	Difference between area	95%CI^d^	*p* Value	Categorical	95%CI	*p* Value	Categorical	95%CI	*p* Value
Total									
PAR^a^ +FIB-4 vs. FIB-4^b^	0.1239	0.0639, 0.1838	<.0001	0.1890	0.1308, 0.2473	<.0001	0.1122	0.0767, 0.1477	<.0001
PNI^c^+FIB-4 vs. FIB-4	0.067	0.0060, 0.1276	.003	0.1236	0.0729, 0.1744	<.0001	0.0513	0.0261, 0.0764	<.0001

^a^Platelet-to-albumin ratio.

^b^Fibrosis-4.

^c^Prognostic Nutritional Index.

^d^Confidence interval.

^e^Net reclassification improvement.

^f^Integrated discrimination improvement.

**Figure 6.**
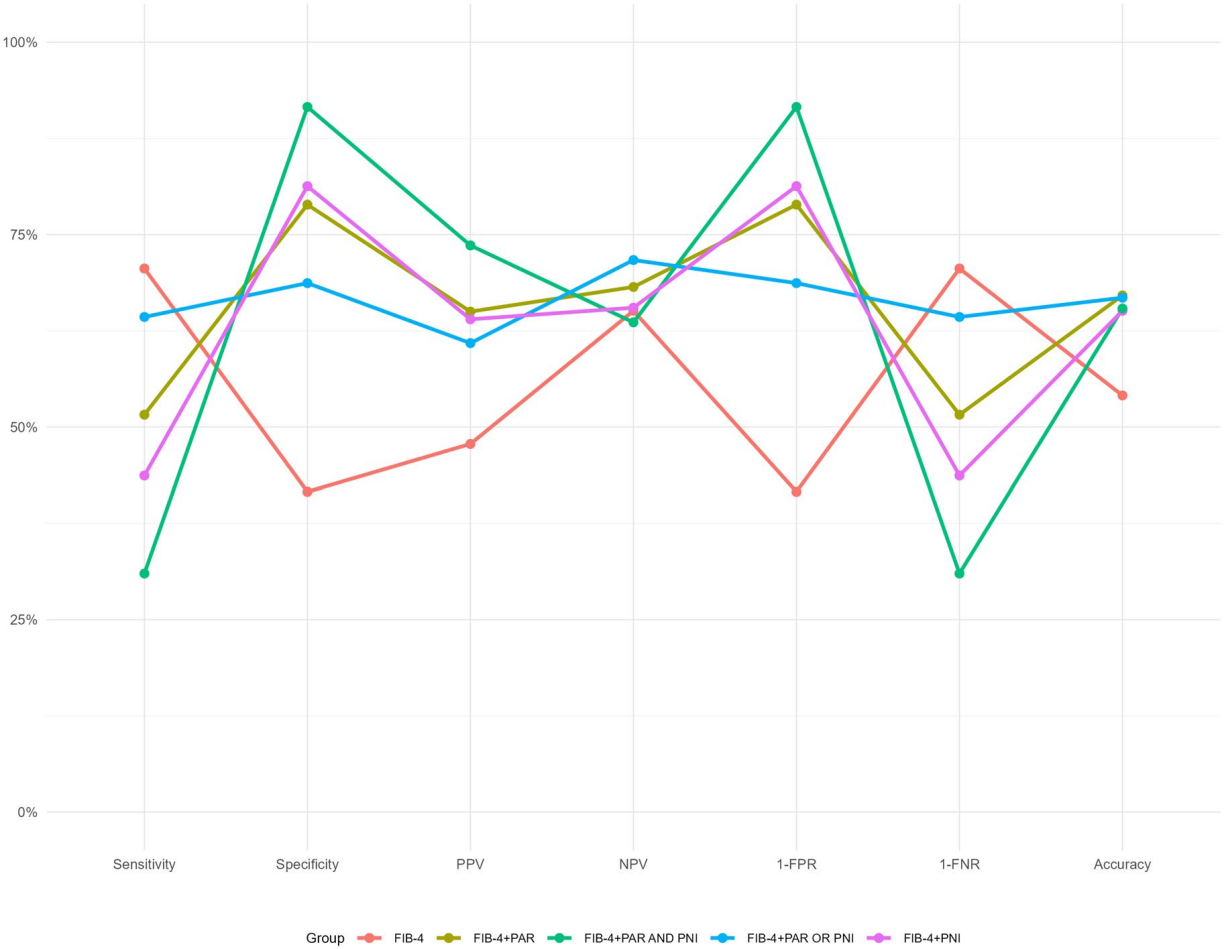
The enhancement of advanced liver fibrosis/cirrhosis diagnostic performance by combining PAR and PNI with FIB-4 in the full cohort.

We next assessed the performance of specific diagnostic strategies that combined FIB-4 with PAR and/or PNI using predefined cut-offs. The results demonstrated that, compared to using FIB-4 alone, the combined strategies significantly optimized multiple diagnostic metrics (Figure 6). Specifically, the combination of FIB-4 and PAR showed notable improvements in specificity (78.9% vs 41.6%), positive predictive value (65.0% vs 47.8%) and overall accuracy (67.1% vs 54.1%). The FIB-4 and PNI combination further increased specificity to 81.3%, with a corresponding decrease in sensitivity to 43.7%. The parallel strategy (FIB-4 + ‘PAR or PNI’) maintained relatively high sensitivity (64.3% vs 70.6%) while achieving balanced specificity (68.7%) and the highest negative predictive value (71.7%). In contrast, the serial strategy (FIB-4 + ‘PAR and PNI’) achieved the highest specificity (91.6%) and positive predictive value (73.6%), but at the cost of a significant reduction in sensitivity (31.0%).

## 4. Discussion

Building upon prior research, this study explores a novel approach. While existing literature focuses on validating biomarkers or developing multiparametric models [19–21], our work adopts a distinct strategy: we optimize rather than replace the widely used FIB-4 index, thereby enhancing its diagnostic performance. This pragmatic approach may improve clinical feasibility. Although nutritional-inflammatory indices have been previously associated with liver disease outcomes, this study first systematically evaluates their diagnostic value for cirrhosis when combined with FIB-4 and quantifies their contribution under diverse clinical decision-making objectives, providing an evidence-based foundation for personalized diagnosis.

Our study demonstrates that nutritional-inflammatory indices remain significantly associated with cirrhosis after adjusting for confounders. For identifying advanced fibrosis/cirrhosis, these indices – excluding HALP – exhibited discriminative ability comparable to or better than FIB-4. However, in practice, no single nutritional-inflammatory index significantly outperformed FIB-4 alone. Notably, combining nutritional-inflammatory indices (PAR and PNI) with FIB-4 improved diagnostic performance of FIB-4 for advanced fibrosis/cirrhosis to varying degrees, supporting tailored combination strategies for specific scenarios. Specifically, a sequential combination of FIB-4, PAR and PNI increased specificity from 38.5% to 95.1%, making it suitable for screening scenarios that prioritize high specificity, especially in settings with limited medical resources, where minimizing false positives is critical to avoid unnecessary invasive procedures and optimize resource allocation. In routine clinical practice, where a balanced trade-off between sensitivity and specificity is desirable to efficiently identify high-risk individuals for further evaluation, the parallel combination (FIB-4 + ‘PAR or PNI’) represents a preferable option. Although this strategy shows slightly lower sensitivity (61.9%) compared to FIB-4 alone (70.6%), it provides better overall performance in specificity, positive predictive value and accuracy, thereby supporting a more balanced diagnostic optimization. Sensitivity analysis suggests the above findings are relatively robust.

Previous studies show that nutritional-inflammatory indices, reflecting systemic nutritional and inflammatory status, are important in various diseases. In oesophageal squamous cell carcinoma, a high PAR (≥ 5.7 × 10^9^) is an independent prognostic factor with predictive power superior to traditional inflammatory markers [22]. In colorectal cancer, incorporating both PAR and the PNI into the prognostic assessment system significantly improves the ability to predict overall survival [23]. For predicting radiation-induced cystitis in cervical cancer, the combination of PAR and the systemic immune-inflammation index enhances model discrimination (AUC 0.774) [24]. In IgA nephropathy, a high PAR is independently associated with disease activity and the risk of end-stage renal disease (hazard ratio = 2.62) [25]. In critical care, the HALP index has been validated as an effective tool for assessing mortality risk. A HALP value ≤9.94 indicates a significantly increased risk of death, with predictive performance comparable to that of the acute physiology and chronic health evaluation II score. Multivariate analysis further confirms that the HALP is an independent predictor of mortality [26]. Ao and colleagues demonstrated that the PNI serves as a robust indicator of disease severity and prognosis in patients with liver cirrhosis. It exhibits high predictive value for mortality in those with decompensated cirrhosis, as evidenced by an AUC of 0.943, with lower PNI levels being significantly associated with poorer outcomes [27]. The research by Yu et al. indicated that a higher baseline PNI is an independent protective factor for hepatic re-compensation in patients with decompensated primary biliary cholangitis, yielding a hazard ratio of 1.11 [28].

In the context of cirrhosis, evaluating the dynamics of these markers is also of significant importance. This is because cirrhosis results from the progressive development of chronic liver disease from multiple aetiologies. Its core pathological changes are not confined to hepatic fibrosis alone but also encompass systemic metabolic disturbances and immune dysregulation [29–31]. A comprehensive assessment of nutritional-inflammatory markers helps provide a more holistic evaluation of a patient’s overall condition, compensating for the limitations of traditional liver function indices. For instance, in cirrhosis with portal hypertension, hypersplenism accelerates platelet destruction, while decreased hepatic synthetic capacity leads to reduced thrombopoietin production, both contributing to thrombocytopenia. Moreover, diminished hepatic synthetic and reserve capacity results in hypoalbuminemia, and lymphopenia is closely associated with cirrhosis-related immune dysfunction [32,33]. Hence, these markers hold promise as effective biomarkers for identifying cirrhosis.

Our study confirms that combining nutritional-inflammatory markers (PAR, PNI) with FIB-4 can optimize diagnostic performance for advanced fibrosis/cirrhosis. This supports our core hypothesis that PAR and PNI can provide independent and complementary pathophysiological information beyond that offered by FIB-4. The underlying mechanism lies in the complex nature of cirrhosis as a systemic disease. FIB-4 is a valuable tool for assessing liver fibrosis risk, as it primarily quantifies hepatocyte injury and its associated haematological consequences. However, the pathophysiological impact of cirrhosis extends far beyond structural changes in the liver. Indeed, systemic metabolic and immune disturbances can emerge even in early-stage cirrhosis [34]. It is within this context that nutritional-inflammatory markers demonstrate their unique value. The PNI, which integrates albumin (reflecting hepatic synthetic function and nutritional status) and lymphocyte count (reflecting systemic immune status), directly quantifies the systemic pathological state of ‘hepatic synthetic dysfunction–malnutrition–immune exhaustion’ [35,36]. The PAR captures haemodynamic changes and nutritional synthetic function through the ratio of platelets to albumin. Therefore, combining FIB-4 with nutritional-inflammatory markers assesses both intrahepatic structural risk and the extrahepatic systemic dysfunction, with diagnostic improvement stemming from this complementary mechanism.

It should be recognized that the optimal cutoff value of the FIB-4 must be considered in conjunction with specific clinical populations, particularly in metabolic dysfunction-associated steatotic liver disease, where both age and metabolic factors may influence its performance[37]. The primary objective of this study is not to propose another set of cutoff values for specific populations but rather to enhance the diagnostic performance of FIB-4 within its existing interpretive framework. Therefore, even in the presence of cutoff heterogeneity, the combination with other biomarkers to improve diagnostic accuracy and reduce diagnostic uncertainty still holds broad applicability.

Our research has limitations. First, the diagnosis of cirrhosis was not universally confirmed by liver histology, the gold standard, making it difficult to completely avoid the potential for misclassification bias. Secondly, although we have attempted to control for some confounding factors, we cannot adjust for all the potential confounding factors due to the availability of the data. Therefore, the interpretation of the results should be cautious and future prospective research needs to collect more comprehensive information to further verify our findings. Thirdly, the single-centre design may limit the representativeness of the research population, and our findings may not be directly extended to other centre

s with different patient characteristics or medical practice models. Future studies are also required to conduct external validation and cost-effectiveness assessment in prospective cohorts.

## 5. Conclusions

In settings where high sensitivity is prioritized for screening, the FIB-4 index may be used alone to minimize the risk of missed diagnoses. In settings where high specificity is the primary goal, a sequential strategy (FIB-4 + ‘PAR and PNI’) is recommended to reduce the false positive rate as much as possible. For routine screening that requires balanced overall diagnostic performance, a parallel strategy (FIB-4 + ‘PAR or PNI’) demonstrates relatively even performance across metrics and offers good feasibility for clinical application.

## Supplementary Material

Supplemental Material

Supplemental Table 1.docx

Supplemental Table 2.docx

Supplemental Figure 2.png

Supplemental Figure 1.png

Supplemental Table 3.docx

## Data Availability

The datasets used and analysed during the current study are available from the first author upon reasonable request.
