# Supplementary material for: Nutritional-inflammatory indices optimize the diagnostic performance of FIB-4 for advanced fibrosis/cirrhosis in patients with benign liver disease
Source: Ann Med. 2026 Mar 13;58(1):2639649. doi: 10.1080/07853890.2026.2639649 (PMC12990267; doi:10.1080/07853890.2026.2639649)
Supplement: Supplemental Material [file IANN_A_2639649_SM9340.docx]

**Supplemental Figure Caption**

**Supplemental Figure 1**. Bar Chart of Between-Group Differences. A. PAR; B. PNI; C, HALP; D, FIB-4.

**Supplemental Figure 2**. The diagnostic performance of FIB-4, PAR, and PNI. A.​ heatmap of diagnostic performance metrics for FIB-4; B. heatmap of diagnostic performance metrics for PAR; C.​ heatmap of diagnostic performance metrics for PNI.
