# Supplementary material for: Nutritional-inflammatory indices optimize the diagnostic performance of FIB-4 for advanced fibrosis/cirrhosis in patients with benign liver disease
Source: Ann Med. 2026 Mar 13;58(1):2639649. doi: 10.1080/07853890.2026.2639649 (PMC12990267; doi:10.1080/07853890.2026.2639649)
Supplement: Supplemental Table 1.docx [file IANN_A_2639649_SM1616.docx]

**Supplemental Table 1.** Calculation formulas of indices

| Indices | Calculation formulas |
| --- | --- |
| PAR | Platelet count / Albumin level |
| PNI | Albumin + 5 × absolute lymphocyte count |
| HALP | Hemoglobin level × Albumin level × Lymphocyte count / Platelet count |
| FIB-4 | (Age x AST) / (Platelet count x $\text{√ALT}$) |

Abbreviations: PAR, Platelet-to-Albumin Ratio; PNI, prognostic nutritional index; HALP, Hemoglobin, Albumin, Lymphocyte, and Platelet; FIB-4, Fibrosis-4; ALT, alanine aminotransferase; AST, aspartate aminotransferase.
