# Supplementary material for: Nutritional-inflammatory indices optimize the diagnostic performance of FIB-4 for advanced fibrosis/cirrhosis in patients with benign liver disease
Source: Ann Med. 2026 Mar 13;58(1):2639649. doi: 10.1080/07853890.2026.2639649 (PMC12990267; doi:10.1080/07853890.2026.2639649)
Supplement: Supplemental Table 2.docx [file IANN_A_2639649_SM1615.docx]

**Supplemental Table 2.** Diagnostic Performance of nutritional-inflammatory indices

| Indicator | AUC | 95%CI | Cut-off Value | Sensitivity | Specificity |
| --- | --- | --- | --- | --- | --- |
| PAR | 0.698 | 0.633-0.763 | 2.332 | 0.452 | 0.869 |
| PNI | 0.658 | 0.590-0.726 | 38.125 | 0.524 | 0.762 |
| HALP | 0.494 | 0.422-0.566 | 47.397 | 0.500 | 0.557 |

Abbreviations: PAR, Platelet-to-Albumin Ratio; PNI, prognostic nutritional index; HALP, Hemoglobin, Albumin, Lymphocyte, and Platelet; AUC, area under the curve; CI, confidence interval.
