# Supplementary figures and images for: Nutritional-inflammatory indices optimize the diagnostic performance of FIB-4 for advanced fibrosis/cirrhosis in patients with benign liver disease
Source: Ann Med. 2026 Mar 13;58(1):2639649. doi: 10.1080/07853890.2026.2639649 (PMC12990267; doi:10.1080/07853890.2026.2639649)

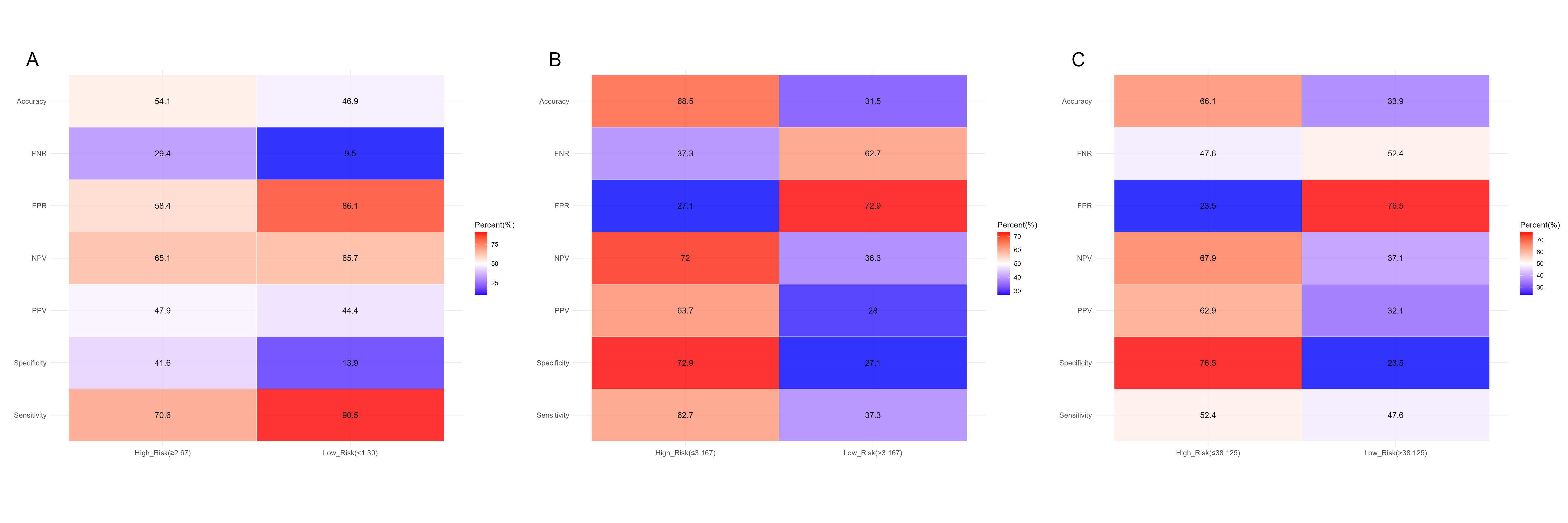

Supplement: Supplemental Figure 2.png [file IANN_A_2639649_SM1613.png]

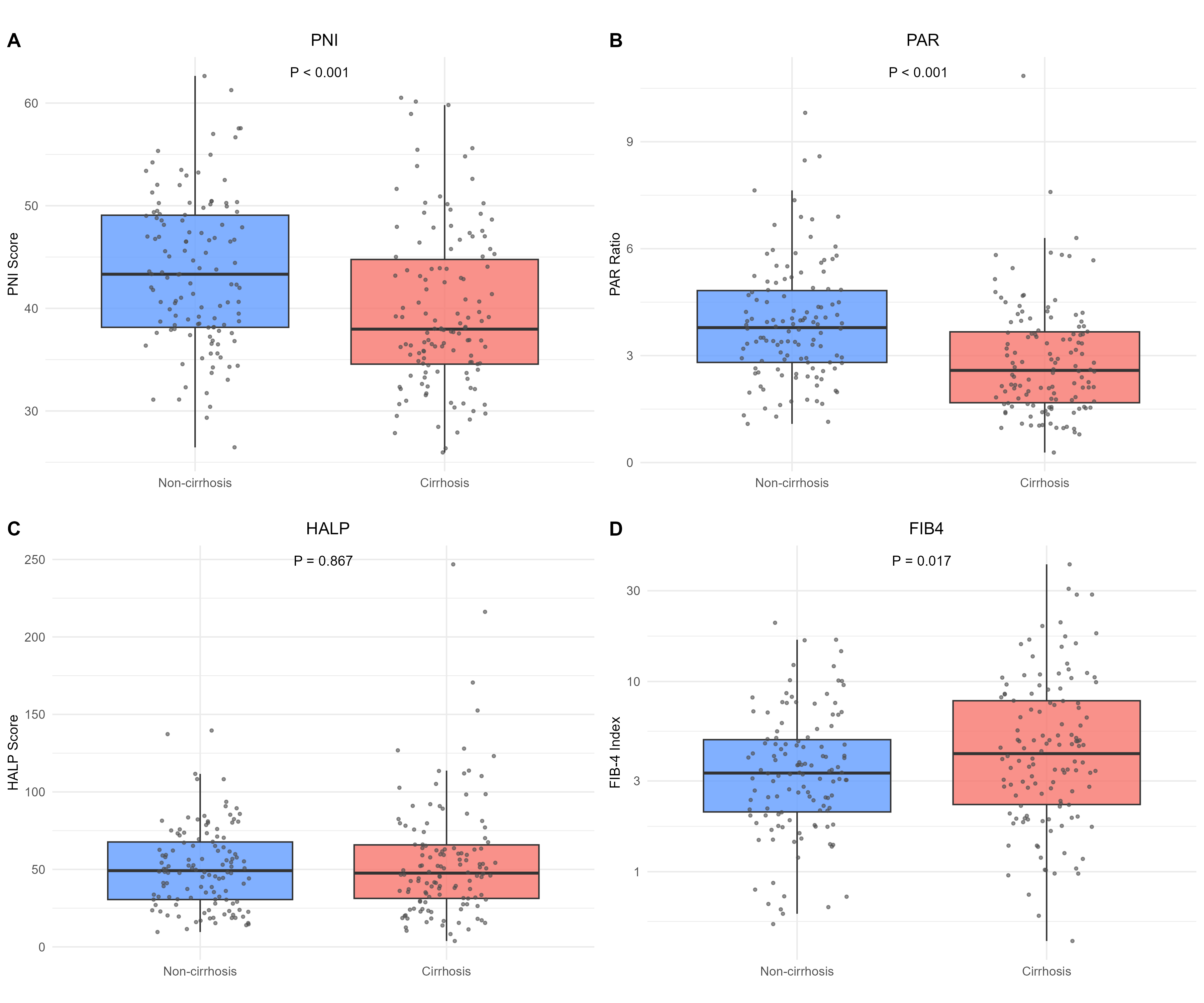

Supplement: Supplemental Figure 1.png [file IANN_A_2639649_SM1612.png]
