# Supplementary material for: Nutritional-inflammatory indices optimize the diagnostic performance of FIB-4 for advanced fibrosis/cirrhosis in patients with benign liver disease
Source: Ann Med. 2026 Mar 13;58(1):2639649. doi: 10.1080/07853890.2026.2639649 (PMC12990267; doi:10.1080/07853890.2026.2639649)
Supplement: Supplemental Table 3.docx [file IANN_A_2639649_SM1611.docx]

**Supplemental Table 3**. Characteristics of participants in the full cohort

| Variable | Non-advanced fibrosis/cirrhosis (N=166) | Advanced fibrosis/cirrhosis (N=126) | P Value |
| --- | --- | --- | --- |
| Age (years) | 48.0 (34.0-56.8) | 55.0 (45.0-61.0) | <0.001 |
| Gender |  |  | 0.906 |
| Male | 126 (75.9%) | 94 (74.6%) |  |
| Female | 40 (24.1%) | 32 (25.4%) |  |
| Etiology |  |  | 0.042 |
| Hepatitis B | 110 (66.3%) | 86 (68.3%) |  |
| Hepatitis C | 3 (1.8%) | 9 (7.1%) |  |
| Other | 53 (31.9%) | 31 (24.6%) |  |
| WBC ^1^ (×10⁹/L) | 5.3 (4.3-6.7) | 5.0 (3.5-6.2) | 0.032 |
| Neutrophil (×10⁹/L) | 3.2 (2.4-4.4) | 3.0 (2.2-4.3) | 0.187 |
| Lymphocyte (×10⁹/L) | 1.3 (1.0-1.7) | 1.0 (0.7-1.4) | <0.001 |
| Monocyte (×10⁹/L) | 0.5 (0.4-0.7) | 0.5 (0.3-0.7) | 0.292 |
| Platelet (×10⁹/L) | 146.0 (110.0-192.8) | 85.5 (60.0-121.8) | <0.001 |
| Hemoglobin (g/L) | 128.5 (108.2-143.0) | 120.0 (104.0-133.0) | 0.004 |
| ALT ^2^ (U/L) | 68.5 (31.2-108.5) | 64.0 (39.2-153.2) | 0.068 |
| AST ^3^ (U/L) | 81.0 (38.2-154.0) | 43.5 (23.0-156.8) | 0.028 |
| Albumin (g/L) | 37.5 (32.9-41.9) | 32.7 (29.0-38.2) | <0.001 |
| TBIL ^4^ (μmol/L) | 129.7 (24.4-295.4) | 63.4 (21.9-230.1) | 0.292 |
| DBIL ^5^ (μmol/L) | 111.2 (15.2-229.4) | 39.5 (11.2-188.0) | 0.164 |
| ALP ^6^ (U/L) | 129.5 (98.0-154.5) | 116.5 (92.5-150.0) | 0.221 |
| γ-GT ^7^ (U/L) | 134.0 (73.0-230.2) | 71.5 (32.2-117.5) | <0.001 |
| PT ^8^ (s) | 15.2 (13.6-19.0) | 17.8 (15.4-21.8) | <0.001 |
| APTT ^9^ (s) | 40.8 (37.7-46.1) | 44.2 (39.6-50.9) | 0.001 |
| PNI ^10^ | 43.5 (38.1-49.5) | 38.0 (34.6-44.8) | <0.001 |
| PAR ^11^ | 4.0 (3.0-5.1) | 2.6 (1.7-3.7) | <0.001 |
| HALP ^12^ | 47.1 (27.3-62.5) | 47.6 (31.3-65.8) | 0.285 |
| FIB4 ^13^ | 3.1 (1.8-4.9) | 4.2 (2.3-7.9) | 0.001 |

^1^ white blood cell; ^2^ alanine aminotransferase; ^3^ aspartate aminotransferase; ^4^ total bilirubin; ^5^ direct bilirubin; ^6^ alkaline phosphatase; ^7^ γ-glutamyl transpeptidase; ^8^ prothrombin time; ^9^ activated partial thromboplastin time; ^10^ prognostic nutritional index; ^11^ platelet-to-albumin ratio; ^12^ hemoglobin, albumin, lymphocyte, and platelet; ^13^ fibrosis-4.
